# Effect of prepartum dietary energy density on beef cow energy metabolites, and birth weight and antioxidative capabilities of neonatal calves

**DOI:** 10.1038/s41598-022-08809-6

**Published:** 2022-03-22

**Authors:** Hao Chen, Chunjie Wang, Simujide Huasai, Aorigele Chen

**Affiliations:** 1 College of Animal Science, Inner Mongolia Agricultural University, Hohhot, 010018 China; 2 College of Veterinary Medicine, Inner Mongolia Agricultural University, Hohhot, 010018 China

**Keywords:** Biotechnology, Cell biology, Biomarkers

## Abstract

The objective of this study was to investigate the effect of prepartum diets that differ in energy density on beef cow energy metabolites and birth weight, immunity and antioxidative capabilities of neonatal calves. On d 0 (approximately 45 d before calving), 90 multiparous Angus cows (BW = 510 ± 16 kg) were randomly allocated into 1 of 9 drylot pens (10 cows/pen). Each pen was randomly assigned to a treatment condition (three pens/treatment), the cows in each treatment were assigned randomly to receive a high-energy (HE) density diet (NEm = 1.67 Mcal/kg of DM), medium-energy (ME) density diet (NEm = 1.53 Mcal/kg of DM), or low-energy (LE) density diet (NEm = 1.36 Mcal/kg of DM). Blood samples were collected − 45, − 21, − 14, and − 7 d from calving, and plasma concentrations of cortisol, glucose, total protein, β-hydroxybutyrate (BHBA), and nonesterified fatty acids (NEFAs) were measured. After calving, the birth weights, body height, body length, thoracic girth and umbilical girth of the calves in each group were recorded, and blood samples were collected for analysis of IgG, IL-2, IL-4, IL-6, total antioxidant capacity, superoxide dismutase, glutathione peroxidase, and maleic dialdehyde levels. The amounts of feed offered and orts were recorded for individual cows 4 d/wk. The results indicated that although dry matter intake (DMI) levels did not differ among the LE, ME, and or HE groups before parturition, the group that received the HE diet had higher plasma glucose concentrations and lower prepartum blood NEFA concentrations than the other groups. Birth weight, body height, thoracic girth, and levels of IL-2, cortisol, total antioxidant capacity, and superoxide dismutase were increased in calves of the HE group compared with those of the LE group. The plasma IL-4 and serum IgG concentrations tended to be decreased in the ME group compared with the HE group, and the ME group had lower maleic dialdehyde concentrations; maleic dialdehyde levels were significantly increased in the LE group compared with the HE group. Overall, these results indicate that feeding of a low-energy diet during the last 45 d before parturition has negative effects on the growth, immunity, and antioxidative capabilities of neonatal calves. Increasing maternal energy density during late gestation may be useful to improve the energy status of cows.

## Introduction

The growth and development of foetuses depend on maternal nutrition, which provides glucose and amino acids to the foetus by blood cycling^1^. About 75% of the fetal weight increase occurs during late gestatioin^2^. Therefore, during this period, beef cows experience an increase in nutrient requirements to maintain body condition and foetal growth. A few studies have reported that changes in maternal nutrition during the prepartum period result in perturbations in foetal growth and metabolism^3,4^. Research has suggested that changes in dietary energy intake influence calf birthweight. Wilson et al. observed greater calf birth weight when cows were fed higher energy diet^5^. Hence, formulating a diet that provides sufficient nutrition is a major management goal for beef producers to increase the performance of cows and calves.

Dry matter intake (DMI) begins to decline in late gestation, the energy requirements of beef cows increase to support foetal growth^6^. Furthermore, the increase in energy demands results in a negative energy balance (NEB), and NEB of gestating beef cows is one of the stressors that can be detrimental for placental environment and calf development^7^. The combined incidence of metabolic disorders resulting from the NEB means that half of all cows calve without health problems^8^. Some researchers have suggested that a higher energy concentration in the precalving diet can improve DMI, increase body condition scores (BCS), and reduce the mobilization of adipose tissue, which could enhance the productive performance of beef cows^9,10^. Cafe et al. reported that chronic nutritional restriction in the precalving that resulted in fetal growth retardation and reduced birth weight by an average of 3.7 kg^11^. In contrast to this viewpoint, others have shown that restricting prepartum energy consumption should result in greater increases in DMI and energy intake postpartum than a higher-energy density diet, which could improve the energy balance of cows^12,13^. Although it is still debatable, both low- and high-energy prepartum diets could have positive effects on cow performance and foetal growth.

Adequate energy supplies during gestation are not only necessary to meet the nutrient requirements of cows but may also benefit the growth performance and metabolism of calves. The effects of energy intake during late gestation on the birth weights and organ development of neonatal calves have been extensively documented, most of the above studies used low or high energy density diets with restricted DMI to control energy intake, but these experimental results do not reflect the true energy intake and metabolism of cows housed in a free-stall barn on commercial farms. Furthermore, several studies evaluated energy restriction during late gestation of beef cows on calf humoral immune response, but few studies have examined the effect of the higher energy density of prepartum diet on the immunity and antioxidative capabilities of neonatal calves^14–16^. The energy balance of pregnant cows and neonatal calves health remain focal points for beef producers and researchers. Therefore, our hypotheses were that (1) a high-energy density diet could reduce mobilization of adipose tissue and (2) a high-energy density diet could improve the growth and health of neonatal calves. The objective of this study was to evaluate the effect of prepartum dietary energy density on beef cow energy metabolites and the birth weights, immunity levels and antioxidative capabilities of neonatal calves.

## Materials and methods

### Ethics approval and consent to participate

All the animal procedures were carried out according to the protocols approved by the College of Animal Science, Inner Mongolia Agricultural University, China. All the experimental animals were approved by the Institutional Animal Care and Use Committee in the College of Animal Science, Inner Mongolia Agricultural University, China. We confirm that the study complied with the Animal Research: Reporting of In Vivo Experiments (ARRIVE) guidelines. Permission along with written informed consent was obtained from the cow owner.

### Animals and treatments

The experimental procedures used were approved by the Institutional Animal Care and Use Committee of Inner Mongolia Agricultural University (Hohhot, China). All methods were carried out in accordance with relevant guidelines and regulations. This study was carried out on a commercial farm. Ninety multiparous Angus cows (BW = 510 ± 16 kg) were selected from a larger breeding group based on foetal ageing determined by transrectal palpation. All cows were fed the same diet prior to the experiment (NEm = 1.53 Mcal/kg of DM). Fixed time artificial insemination (FTAI) was performed in this study, semen from nine Angus sires were randomly assigned to each cow on the same day, each of the treatments receive 3 of the 9 Angus bulls. Cows were grouped according to body weight (BW) and expected calving date and were then randomly allocated into nine pens in a drylot feeding facility (n = ten cows/pen).

Each pen was randomly assigned to a treatment condition (three pens/treatment), the dietary energy restriction/feeding level was based on NRC. The % of gestational energy requirements was 110% (HE), 100%(ME) and 90% (LE), respectively, and the diets were formulated to the same nitrogenous. The cows in each treatment were assigned randomly to receive a high-energy (HE) density diet (NEm = 1.67 Mcal/kg of DM), medium-energy (ME) density diet (NEm = 1.53 Mcal/kg of DM), or low-energy (LE) density diet (NEm = 1.36 Mcal/kg of DM), and were fed these treatment diets from day 45 before parturition, until parturition. Cows in each group calved on three adjacent days. Dry straw, which was the first batch of materials loaded into the TMR mixer, was chopped into small particles and then mixed with concentrate and corn silage. Different amounts of water were added to each of the three diets to adjust the DM content to between 48 and 50%. The cows were housed in a free-stall barn with a delivery room and were offered the TMR twice daily (at 0800 and 1500 h) and given unlimited access to fresh water.

### Sample collection

The amounts of diet DM that was offered and refused were obtained daily for each pen by drying diet samples that were offered and refused in a forced-air oven at 56 °C for 48 h. Daily DMI was determined by subtracting the daily diet DM refused from the daily diet DM offered. Samples of total mixed diet offered to the cows were collected weekly and were then sent in duplicate to the laboratory for chemical analysis of all nutrients (Table 1). The samples were analysed for concentrations of CP (CP, method 984.13)^17^, neutral detergent fibre (NDF) and acid detergent fibre (ADF) according to^18^. NEm values were calculated using equations from the NRC^19^. BW was measured at d − 45 and − 1 d relative to expected parturition. Each weighing of the cows in each group is carried out after 12 h of feeding. BCS was assessed independently by three individuals on a 1 to 9 scale^14^.

**Table 1:** Average weekly chemical composition of total mixed diets (HE, ME and LE) offered to mature cows during the last 45 d of gestation (d 0 to calving).

Item	Group^a^		
	HE	ME	LE
**Ingredient, % of DM**			
Corn silage	42.0	33.0	24.0
Dry rice straw	18.0	27.0	36.0
Corn	22.8	15.6	9.3
Wheat bran	5.2	12.1	15.8
Soybean meal	2.4	2.4	3.6
Cottonseed meal	3.6	5.1	6.8
CaHPO_4_	0.6	0.6	0.6
NaHCO_3_	0.4	0.4	0.4
NaCl	0.5	0.5	0.5
Unifat^b^	2.5	1.3	0.0
Premix^c^	2.0	2.0	2.0
Total	100	100	100
**Nutrient composition**			
CP, % of DM	11.55	11.78	11.69
ADF, % of DM	23.52	26.88	28.93
NDF, % of DM	37.51	41.62	44.30
TDN, % of DM	71.19	68.31	66.56
NEm,^d^ Mcal/kg DM	1.67	1.53	1.36
Ca, % of DM	0.72	0.79	0.75
P, % of DM	0.36	0.34	0.42

^a^HE = high energy (NEm = 1.67 Mcal/kg of DM); ME = medium energy; (NEm = 1.53 Mcal/kg of DM); LE = low energy (NEm = 1.39 Mcal/kg of DM).

^b^Fractionated palm fatty acids (China Benefit Agriculture, Beijing, China).

^c^Premix contained (per kg of premix): 480,000 IU of vitamin A, 90,000 IU of vitamin D_3_, 3,500 IU of vitamin E, 2400 mg of Fe, 168 g of Ca, 38 g of P, 950 mg of Cu, 1,500 mg of Mn, 3,150 mg of Zn, 28 mg of I, 33 mg of Se, and 26 mg of Co.

^d^NEm was calculated based on NRC (2001).

Blood samples (5 mL) were collected from all cows via jugular venipuncture into sodium heparin-containing tubes for plasma harvest after 12 h of feed and water withdrawal − 45, − 21, − 14, and − 7 d from calving to determine the plasma concentrations of cortisol, glucose, total protein, BHBA, and NEFA. Blood samples were immediately placed on ice following collection and were then centrifuged at 2,000×*g* for 25 min at 4 °C. The plasma samples aspirated from the collection tubes and moved to new tubes before stored at − 20 °C until laboratory analysis.

Less than 24 h postpartum, the birth weight and body height, body length, thoracic girth and umbilical girth of each calf were recorded. The number of newborn male calves in the HE, ME and LE groups are 13, 20 and 15 respectively. All measurements were taken when each calf was standing naturally with its head raised and weight was distributed on all four feet. Blood samples (5 mL) were collected from all calves via jugular venipuncture into sodium heparin-containing tubes within 4 h of birth; the calf blood samples were handled in a similar fashion as the cow blood samples, and the plasma was stored at − 20 °C for analysis of IL-2, IL-4, IL-6, total antioxidant capacity (T-AOC), maleic dialdehyde (MDA), glutathione peroxidase (GSH-Px), and superoxide dismutase (SOD) levels. Additional blood samples (5 mL) were collected after 24 h of birth, from the jugular veins of all calves into tubes containing no additives for serum harvest to evaluate serum concentrations of IgG. Plasma and serum samples were stored at − 20 °C until laboratory analysis.

### Laboratory analyses

Plasma cortisol, glucose, total protein, interleukin-2 (IL-2), interleukin-4 (IL-4), interleukin-6 (IL-6), BHBA, and total serum immunoglobulin G (IgG) concentrations were determined using commercially available bovine ELISA kits (Baoman Biological, Shanghai, China) according to the manufacturer’s protocol. According to the manufacturer’s instructions, a commercial colorimetric assay kit (Nanjing Jiancheng Bioengineering Institute, Nanjing, China) was used to measure the activities of glutathione peroxidase (GPx), superoxide dismutase (SOD), and malondialdehyde (MDA), total antioxidant capacity (T-AOC), and BHBA and NEFA concentrations in the plasma. The samples were not performed in duplicate or triplicate.

In the NEFA assay, the stop solution changed the colour of the assay from blue to yellow, and the intensity of the colour was measured at 450 nm using a spectrophotometer (UV-1780 Shimadzu, Japan). To measure the NEFA concentrations in the samples, the NEFA ELISA Kit included a set of calibration standards. The calibration standards were assayed at the same time as the samples, allowing the generation of a standard curve of optical density versus NEFA concentration. The NEFA concentrations in the samples were then determined by comparing the O.D. of the samples to the standard curve.

### Statistical analysis

One cow gave birth to a dead foetus and did not complete the study. Thus, 89 cows (HE, n = 30; ME, n = 30; and LE, n = 29) completed the experiment and were used in the analysis.

All data were analysed in a completely randomized design using the MIXED procedure of SAS (SAS Inst. Inc., Cary, NC; version 9.3) and the Satterthwaite approximation to determine the denominator degrees of freedom for tests of fixed effects^15^. Daily cow intake data were pooled by week to simplify data analyses, interpretation, and reporting. Daily cow intake of diet DM, CP, and NEm were analysed as repeated measures and were tested for the fixed effects of cow gestational diet, week of study, and resulting interactions, using pen (treatment) as the subject^15^. The levels of different factors in the blood of cows were analysed as repeated measures and tested for fixed effects of cow gestational diet, day of the study, and resulting interactions. The REPEATED procedure with the first order autoregressive covariance structure was used for variables repeatedly measured over time. All calf data were assessed according to the general linear model (ANOVA) procedure of SAS (SAS Inst. Inc., Cary, NC; version 9.3). Duncan’s test was used to identify significant differences among mean values. Calf gender were included as categorical variable in analyses but were removed from the model when P > 0.10. Percentage of male calves at birth was tested for fixed effects of cow gestational diet using GLIMMIX procedure of SAS. The data are presented as the means ± standard errors, and a value of P < 0.05 was set as the significance level.

## Results

### DMI and plasma metabolites

The main effects of precalving energy density, time, and their interactions on DMI, NEm intake, CP intake, and plasma metabolites are summarized in Table 2. The total average prepartum DMI was not affected by the reduced-energy density diets, but the NEI of the LE group was lower than the NEI of the HE group (P < 0.05). By design, cows had a similar overall CP intake (P = 0.59) (Table 2). Effects of treatment × day of study and treatment were not detected (P ≥ 0.25) for plasma concentrations of total protein and BHBA of HE, ME and LE cows (Table 2). Plasma glucose concentrations in the HE group were significantly higher than those in the LE and ME groups. However, the plasma NEFA level was decreased (P < 0.05) in HE and ME cows compared to LE cows.

**Table 2:** Precalving intake and plasma measurements of late gestating beef cows offered isonitrogenous, total-mixed diets formulated to provide 110 (HE), 100 (ME) or 90% (LE) of daily NEm requirements during the last 45 d of gestation.

Item	Group^1^			SEM	Treatment	*P*-value
	HE	ME	LE			Treatment × time
**Cow intake**						
BW d 0, kg	517	505	509	16	0.59	
BW before calving, kg	573	558	546	23	0.21	0.36
BCS d 0	5.72	5.71	5.68	0.08	0.86	
BCS before calving	6.05^a^	5.89^ab^	5.81^b^	0.12	0.03	0.29
Total DMI, kg/d	10.82	10.71	10.48	0.19	0.73	0.86
NEm, Mcal/d	18.03^a^	16.37^ab^	14.46^b^	0.33	0.021	0.26
CP, kg/d	1.25	1.22	1.23	0.058	0.59	0.32
**Cow serum measurements**^**2**^						
Glucose, mmol/L	4.86^a^	4.18^ab^	3.75^b^	0.31	0.025	0.53
Total protein, mg/mL	155.18	152.59	148.94	2.17	0.66	0.40
BHBA, μmol/L	377.68	352.18	365.80	5.69	0.48	0.25
NEFA, μmol/L	426.05^b^	448.47^b^	566.31^a^	49.38	0.019	0.22
Cortisol, ng/mL	18.69	19.55	21.33	0.22	0.08	0.17

^1^HE = high energy (NEm = 1.67 Mcal/kg of DM); ME = medium energy; (NEm = 1.53 Mcal/kg of DM); LE = low energy (NEm = 1.39 Mcal/kg of DM).

^2^Overall plasma concentrations of glucose, total protein, BHBA, NEFA, and cortisol collected on d 0, 24, 31, and 38.

^a,b^^,c^Means bearing different superscripts in the same row difer significantly (P < 0.05).

### Calf weights and measurements

The weights and measurements of neonatal calves in the HE, ME, and LE groups tended to increase linearly with increasing energy density (Table 3). Calf weight (P < 0.05), body height (P < 0.05), and thoracic girth (P < 0.01) in the LE group were significantly lower than those in the HE group.

**Table 3:** Effects of prepartum maternal energy density on birth weights and measurements in neonatal calves.

Item	Group^1^			SEM	*P*-value
	HE	ME	LE		
Birth weight, kg	39.62^a^	37.17^ab^	33.68^b^	3.53	0.039
Bulls birth weight, kg	41.88^a^	37.92^ab^	34.13^b^	4.62	0.020
Heifers birth weight, kg	37.63	35.84	33.28	2.69	0.156
Body height, cm	73.97^a^	69.59^a^	62.28^b^	1.96	0.033
Body length, cm	73.23	70.63	71.07	2.11	0.37
Thoracic girth, cm	81.94^a^	76.55^b^	67.52^c^	2.63	0.002
Umbilical girth, cm	72.52	73.90	69.36	1.78	0.61

^1^HE = high energy (NEm = 1.67 Mcal/kg of DM); ME = medium energy; (NEm = 1.53 Mcal/kg of DM); LE = low energy (NEm = 1.39 Mcal/kg of DM).

^a,b^^,c^Means bearing different superscripts in the same row difer significantly (P < 0.05).

### Immunity capabilities of neonatal calves

The effects of prepartum maternal energy density on IL-2, IL-4, IL-6, cortisol, and serum IgG levels in neonatal calves are presented in Table 4. Concentrations of IL-2, IL-4 and cortisol in neonatal calves were strongly influenced by maternal energy density during the last 45 d of pregnancy such that the concentrations of IL-2 (P < 0.01), IL-4 (P < 0.05) and cortisol (P < 0.05) were much lower in the LE group than in the HE group. IL-6 and serum IgG concentrations did not differ among groups.

**Table 4:** Effects of prepartum maternal energy density on the serum immune indexes in neonatal calves.

Item	Group^1^			SEM	*P*-value
	HE	ME	LE		
IL-2, ng/mL	5.87^a^	4.96^b^	4.10^c^	1.39	0.001
IL-4, ng/mL	1.36^a^	1.22^ab^	1.03^b^	0.28	0.043
IL-6, pg/mL	218.51	201.88	225.19	69.37	0.39
Cortisol, ng/mL	51.37^b^	65.80^a^	72.58^a^	6.56	0.033
Serum IgG, mg/dL	3,898^a^	3,315^ab^	3,322^ab^	517	0.07

^1^HE = high energy (NEm = 1.67 Mcal/kg of DM); ME = medium energy; (NEm = 1.53 Mcal/kg of DM); LE = low energy (NEm = 1.39 Mcal/kg of DM).

^a,b^^,c^Means bearing different superscripts in the same row difer significantly (P < 0.05).

### Antioxidative capabilities of neonatal calves

The effects of prepartum energy density on antioxidation capability in neonatal calves are shown in Table 5. Plasma T-AOC levels in neonatal calves were greatly decreased in the LE group compared with the HE group (P < 0.01). SOD concentrations in the LE group were lower (P < 0.05) than those in the HE and ME groups (P < 0.05), whereas the plasma MDA concentrations of calves in the LE and ME groups were significantly higher than those of calves in the HE group (P < 0.05). Consequently, the antioxidative capabilities of neonatal calves were altered by maternal energy density during the last 45 d of gestation.

**Table 5:** Effects of prepartum maternal energy density on the antioxidation capability in neonatal calves.

Item^2^	Group^1^			SEM	*P*-value
	HE	ME	LE		
T-AOC,U/mL	0.63^a^	0.52^ab^	0.33^b^	0.12	0.001
GSH-Px,U/mL	3.89^a^	3.66^a^	3.16^ab^	6.93	0.085
SOD,U/mL	117.51^a^	111.89^a^	92.14^b^	0.87	0.016
MDA, nmol/mL	2.11^b^	2.96^a^	3.31^a^	1.05	0.027

^1^HE = high energy (NEm = 1.67 Mcal/kg of DM); ME = medium energy; (NEm = 1.53 Mcal/kg of DM); LE = low energy (NEm = 1.39 Mcal/kg of DM).

^2^*T-AOC* total antioxidant capacity, *GSH-Px* glutathione peroxidase, *SOD* superoxide dismutase, *MDA* malondialdehyde.

^a,b^^,c^Means bearing different superscripts in the same row difer significantly (P < 0.05).

## Discussion

The factors that regulate DMI are complex and encompass breed in addition to nutrient and management factors^20^. The average DMI for the three treatment groups was 10.4–10.8 kg/d prepartum, and we observed that changes in prepartum dietary energy density resulted in no differences in DMI. The HE diet supplies more net energy per kg than the ME and LE diets, which caused HE group cows to have increased net energy intake. Other studies have also confirmed that cows fed a high-energy–density diet during gestation easily consume more energy if the DMI is similar^21,22^.

Maternal metabolic products can provide essential nutrients for foetuses and lead to promotion of foetal tissue growth^23^. Late gestation is a critical time since foetal growth and development occur during the last 2 trimesters of gestation^15^. Therefore, beef cows experience increased energy requirements to ensure proper foetal growth. Tanner et al. (2020) fed multiparous beef cows with the same basal forage diet or a diet that was supplemented with 0.2% BW of dry-rolled corn (DM basis) from d 110 to 265 of gestation^24^. It was found that the calves of the supplemented cows were heavier than those of the basal forage diet-fed cows at 3 wk postpartum. The effects of maternal nutrient restriction or adequate nutrient supply during late gestation on the performance of newborn calves have been extensively documented. However, few studies have reported that changes in dietary energy during late gestation do not influence birth weight. In the current study, male calves born to cows fed the HE diet during late gestation were 7.7 kg heavier (P < 0.01) at birth than those born to cows fed the LE diet. About a 10% increase in the weights was observed in Holstein neonatal calves of dams who were fed high-energy diets during the last 21 d before parturition^25^.

Dietary energy levels during pregnancy can change maternal metabolic status and lead to disturbed partitioning of foetal substrates^8^. In this study, the plasma concentrations of Glu and NEFAs in beef cows were affected by energy density during the last 45 d of gestation. It is likely that HE diet preparations reprogramme the supply of nutrients from cows to foetuses, which results in higher birth weights.

Interestingly, sex-specific effects on the birth weights of neonatal calves were observed when birth weight data were analysed separately for bull and heifer calves. The birth weights of bull calves were decreased by reduced-energy density diets, while no differences in the birth weights of heifer calves were observed among treatment groups, with an average of 35.65 kg across treatments. A recent study indicated the similar sex-specific effects on the birth weights of progeny after beef cows were fed a high- or low-fat diet during the last 2 trimesters of gestation^26^. In addition, bull calves born to cows fed a high CP diet had higher birth weights than those bull calves who came from cows fed a low CP diet, and no differences were observed among treatments in the birth weights of heifer calves^27^. However, the reason for this sex-specific effect of feeding HE diets during late gestation on the birth weights of neonatal calves is not clear. The efficiency of the placenta was greater for female foetuses than for male foetuses in the presence of dietary changes^28^. The diet-dependent adaptation capacity of the placentas of female foetuses is attributed to sexual dimorphism in placental DNA methylation^29^. This increased placental efficiency may be important in explaining the difference in the birth weights of male and female calves in response to prepartum dietary energy levels in the current study.

Beef cows are prone to peripartum metabolic disorders; thus, it is important to assess nutritional status by determining metabolic profiles. Blood Glu can be used as an indicator of the energy metabolism of cows, as it changes moderately in blood^30^. A previous study demonstrated that ad libitum intake to supply 150% of prepartum energy requirements resulted in higher prepartum blood Glu levels as compared to restriction of intake^13^. In the present experiment, blood Glu concentrations were greater in HE diet-fed cows than LE-fed cows. This may be because the HE diet contained highly fermentable starch-rich energy sources, which led to increased blood Glu concentrations via propionate conversion in the liver^15^. Interestingly, we found that Glu concentrations were not different between ME and LE cows. Further investigations into the interaction between prepartum energy intake, energy metabolism, and blood Glu concentrations in beef cows are warranted. Its observed that an imbalance between reduced dry matter intake and high energy requirements during late gestation leads to mobilization of fat from adipose tissue and increase plasma NEFA concentrations^31^.

Plasma NEFA levels increased upon supplementation with 0.2% BW of dry-rolled corn during mid- to late-gestation in Angus breeding cows^25^. In the present study, LE cows mobilized more lipid stores, and their plasma NEFA concentrations increased, which suggests that HE cows exhibit less fat mobilization and indicate that feeding a high-energy diet results in an increased energy status in late gestation cows. A recent study have indicated that supplementation of dry-rolled corn at 0.2% of BW during mid- to late-gestation decreased plasma NEFA concentration^24^. In this study, plasma BHBA levels were not influenced by a change in maternal energy density during the last 45 d before parturition. The reasons for the discrepancies in the effects of prepartum maternal energy density on BHBA concentrations between studies are not clear but include many factors, such as energy requirements, animal breed, age, and body condition^32^.

Transfer of serum immunoglobulins from the mother to colostrum in beef cattle begins 28 d before parturition^14^. Recent study reported similar serum IgG concentrations in beef calves born to cows fed 70% or 100% of NEm requirements during the last 40 d of gestation^33^. In our experiment, the IgG concentrations in serum in the three treatment groups were above the minimum threshold considered adequate for passive immunity transfer (> 1,600 mg/dL)^34^. In addition, we observed a trend of higher (P = 0.07) serum IgG concentrations in neonatal calves from the HE group compared with LE neonatal calves. Our results are in agreement with previous research, which indicated that serum immunoglobulin concentrations decreased in calves born to nutrient-restricted dams^35^. IL-2 plays a vital role in T-cell proliferation^36^. In the present study, IL-2 and IL-4 production in neonatal calves was significantly increased (P < 0.01) in the HE group compared with the LE group during the last 45 d of gestation, which implies that the immune capabilities of neonatal calves may be influenced by prepartum energy density. IL-4 enhances immunoglobulin synthesis and induces the proliferation and differentiation of B cells^37^. Therefore, it is possible that in our study, alteration of prepartum energy density during late gestation affected the production of IL-4, and can contribute to compromised immunoglobulin synthesis.

Cortisol is a naturally occurring glucocorticoid that regulates immune responses and is released after an acute-phase response^38^. Serum cortisol concentrations in calves peak at birth during periparturient processes^39^. In the current study, cortisol concentrations significantly decreased (P < 0.05) in HE group calves, which is in accordance with studies that have indicated that serum cortisol concentrations increase in calves born to cows that are nutrient-restricted during late gestation^40^. Previous studies have shown that cortisol may stimulate an acute-phase response that impairs the innate and humoral immune responses^41,42^. Hence, it is possible that increased prepartum energy density during late gestation affects cortisol production and permanently improves the capacity of offspring to cope with stressors^43^.

Maternal energy density affected the antioxidative capabilities of neonatal calves. In general, the HE group calves had higher levels of T-AOC and SOD than the LE group calves. Oxidative stress occurs as a consequence of an imbalance between prooxidant status and antioxidative defence and changes in the activities of enzymes, such as SOD and GSH-Px^44^. A previous study have showed that the activities of SOD and GSH-Px are affected by nutrition levels^45^. The antioxidative activity of neonatal calves could be significantly affected by poor maternal energy density during the last 3 weeks of gestation^26^. In this study, SOD concentrations were decreased in the LE group, and the higher MDA concentrations in calves of the LE group indicated that oxidative stress was induced in those neonatal calves. The tendency of decreased GSH-Px activity may have been responsible for the reduction in T-AOC levels in the LE group, which may have been due to the occurrence of oxidative stress in the LE group. Overall, the antioxidative capabilities of neonatal calves could be significantly increased by increasing the energy level of cows during the last 45 d of gestation, which may improve the health of neonatal calves.

## Conclusions

In the present study, increasing maternal energy density during the last 45 d of pregnancy resulted in increased DMI and decreased NEFA concentrations, did not influence total DMI, and increased NEI and Glu concentrations, which suggest that feeding a high-energy diet resulted in improved energy status in cows. Compared with a prepartum high-energy density diet, a precalving LE diet was associated with decreased male calf birth weight, body height; decreased IL-2, IL-4, T-AOC, and SOD concentrations; and increased MDA concentrations. We concluded that high maternal energy density at 45 d prepartum may be beneficial for the energy status of cows and may lead to improved postnatal growth and calf health.

## Data Availability

The data sets used and/or analyzed during the current study are available from the corresponding author on reasonable request.
